# ‘How can you punish a child for something that happened over a year ago?’ The impacts of COVID-19 on child defendants and implication for youth courts

**DOI:** 10.1177/00220183231172432

**Published:** 2023-05-21

**Authors:** Samuel Larner, Hannah Smithson

**Affiliations:** Manchester Centre for Youth Studies, 5289 Manchester Metropolitan University, UK

**Keywords:** COVID-19, youth courts, empirical research, Crown Prosecution Service, defence solicitors, children, legal advisors

## Abstract

The project on which this paper is grounded is the first in-depth empirical study of the impacts of COVID-19 on each stage of the English and Welsh Youth Justice System. We take the notion of a child's right to a fair trial as the lens by which we detail the findings from our research. The paper documents the experiences of professionals working in the courts and children who had contact with the courts during the pandemic. While we concentrate on processes in England and Wales as an exemplar of the impact of COVID-19, recognising that globally, courts were experiencing similar challenges, initiates a discourse about how to re-envision their role in wider criminal justice systems in a COVID-19 world. Our research demonstrates an urgent need for renewed consideration of what support children need to effectively participate in court, and where and how children's cases should be heard. The pandemic demonstrated that creativity is possible and creates a timely opportunity to review the evidence and think more radically about a welfare-based, trauma-informed court process for children.

## Introduction

The project on which this paper is grounded is the first in-depth empirical study of the impacts of COVID-19 on *each* stage of the English and Welsh Youth Justice System. The Greater Manchester (GM) region of North-West England served as a case study area (as part of The Greater Manchester Youth Justice University Partnership (GMYJUP)), and we additionally drew heavily on national literature and in-depth interviews with national stakeholders from across the youth justice sector.^1^

This paper will focus on the key findings to emerge from the adaptations made to youth courts during the pandemic. We draw on findings from interviews across the GM region with defence solicitors, Crown Prosecution Service (CPS) staff, legal advisors, Youth Offending Team (YOT) professionals, children working with YOTs and children in custody. It is our intention that this paper will document the experiences of professionals working in the courts and children who had contact with the courts during the pandemic. We emphasise both the benefits and the challenges of the adaptations made to Youth Courts. While we concentrate on processes in England and Wales as an exemplar of the impact of COVID-19, recognising that globally, courts were experiencing similar challenges, initiates a discourse about how to re-envision their role in wider criminal justice systems in a post-COVID-19 world.

## The Impact of COVID-19 on Courts

Baldwin et al. note that few courts were prepared for a pandemic.^2^ A paper by Sourdin et al. provides an overview of global court processes.^3^ There are striking similarities across continents. For instance, a move to reduce the numbers of cases heard, scaling back cases heard in physical courtrooms, and conducting hearings remotely through remote platforms. For the first time in its history, the Supreme Court of the United States used remote technology to enable its functionality.^4^

In their review of the literature, Harris and Goodfellow detail that almost half of all court buildings in England and Wales were closed in March 2020 as the United Kingdom entered a national lockdown.^5^ Jury trials were suspended in March 2020 and re-introduced in May 2020 and much court business moved online and cases were stalled. However, the courts did not suspend business completely: Magistrates’ Courts initially heard only urgent work (e.g., overnight police custody cases and cases where a child was remanded in the secure estate). The Ministry of Justice (MoJ) and Her Majesty's Courts and Tribunals Service (HMCTS) moved swiftly in March 2020 to keep 157 priority court and tribunal buildings open for essential face-to-face hearings. This represented 42% of the 370 Crown, Magistrates, County and Family courts and tribunals across England and Wales.^6^ From mid-April 2020, the senior judiciary instructed courts to prioritise listings according to three priority categories. The highest priority included urgent custody cases and the second included serious and time-sensitive youth cases, such as ‘where delay might mean a relevant age-threshold was crossed’.^7^ In July 2020, HMCTS published the ‘Court and Tribunal Recovery Update’, which included the announcement of £142 million pounds to fund improvements to courts including improving remote capabilities.^8^ An updated recovery plan was published in September 2020 setting out plans for more Nightingale Courts, more staff and more video technology, backed by £80 million funding.^9^

A Criminal Justice Joint Inspection (CJJI) Report published in January 2021 provides an overview of the impact of the pandemic on the criminal justice system. The Inspectors note that by April 2020, HMCTS had provided courts with a cloud-based video platform (CVP). By May 2020 CVP had been introduced in 34 Magistrates’ courts and 12 Crown Court centres, and more than 2000 hearings in the Magistrates’ courts and Crown Court had taken place using CVP.^10^ The Inspectorates note a significant decline in its use from its inception – only 15% of CPS cases were CVP applications by September 2020. A combination of court closures and the reduced use of CVP led to significant backlogs in a court system that was experiencing a growth in the backlog of cases prior to the pandemic. For instance, by December 2020, the total live CPS post-charge caseload was 67% higher than the pre-Covid baseline. The Magistrates’ Court live caseload was 83% higher and the Crown Court live caseload was 44% higher.^11^ On 14 December 2020, HMCTS Crown Court cases had increased to more than 53,000.^12^ Indeed, the CJJI conclude that, ‘Our greatest concern, however, remains the situation in courts, and the consequential effect this has on all our inspected sectors’.^13^

We have written extensively elsewhere about the proliferation of needs experienced by justice-involved children during the COVID-19 pandemic.^14^ These additional challenges included a lack of services provided by YOTs, increased exposure to abuse and neglect, solitary confinement in the custodial estate and, relevant to the focus of this paper, their challenging experiences of the court system. We take the notion of a child's right to a fair trial as the lens by which we detail the findings from our research. Lynch and Liefaard note that the upholding of children's rights in ‘normal times’ is a perennial threat for children in justice systems and during the pandemic this threat was magnified.^15^ Despite the fallout from the pandemic having arguably had the most significant impact on children, governments across the globe failed to prioritise them in their responses.^16^ Prior to detailing our findings, we will provide a short history of the genesis of youth courts followed by an overview of youth court responses to the pandemic. McHardy observes that over the first 25 years of the twentieth century, there was a global shift to create separate legal processes for children, including the emergence of youth courts.^17^

The underpinnings of youth courts were non-adversarial and followed a welfare approach as the model by which children would be dealt with by the court.^18^ Under Article 40 (2) of the United Nations Children's Rights Convention (UN CRC) and Art. 6 of the European Convention of Human Rights (ECHR) children have the right to a fair trial, including the right to present a defence and to effective participation in the criminal process.^19^ Children should be treated differently to adults in court, and require person-centred approaches tailored to the individual needs of a child to effectively exercise their right to a fair trial.^20^ This could include recognition of ethnic heritage, special educational needs (SEND), mental health illness and other individual vulnerabilities. By way of context, in England and Wales, when a child (aged between 10 and 17) is charged with an offence, they will appear before a youth court. Youth courts are described as a type of Magistrates’ court and while mainly housed in Magistrates’ courts, they are less formal than Crown Courts. Members of the public are not allowed into the court (unless they have permission). They have either three Magistrates or one District Judge, and do not have a serving jury.^21^

Earlier we detailed the responses and adaptations made by court systems across the globe. We noted that much of this involved reducing the numbers of cases coming to court and pivoting to remote platforms to undertake trials. We are interested in the impact of these responses and adaptations on children. Taking England and Wales as an exemplar, we move to detail the responses of the youth court during the COVID-19 pandemic.

In April 2020, the CPS issued its interim Case Review Guidance to be used by prosecutors during the pandemic. It stated,

All cases involving youth offenders must be dealt with expeditiously and avoid delay, which has at its core the principle that there is little point in conducting a trial for a young offender long after the alleged commission of an offence when the offender will have difficulty in relating the sentence to the offence. To maximise the impact n the youth offender, the case must be dealt with as soon as possible.^22^

Harris and Goodfellow note that youth court data was routinely excluded from the regular data published on hearings and trials during the pandemic.^23^ However, we know that the pandemic caused significant court back-logs, which were already felt acutely in youth justice systems pre-pandemic, with long delays in children's cases coming to court.^24^ The temporary closure of courts due to the pandemic has lengthened delays in cases coming to court. For example, in England and Wales, by the end of June 2020, Her Majesty's Crown Prosecution Inspectorate HMCPSI (2021) report the back-log of children awaiting court had increased by 55% compared with the same period in the previous year.^25^ The back-log of children awaiting court increased at a lower rate than the overall court backlog, which increased by 70%.

We provided details earlier in this paper about the global move to conducting court hearings remotely. The literature indicates that criminal justice professionals in England and Wales felt that remote hearings prevented defendants from participating effectively.^26^ The ability to monitor children and their ability to participate in their own hearing feeds into the larger issue of effective participation: The ability to understand and be involved in what is happening in court. Organisations including NACR0, The Youth Justice Legal Centre, the Royal College of Psychiatrists and the Justice Select Committee have expressed concern and caution about the use of remote court hearings, citing the impact on a child's right to a fair trial and their ability to participate.^27^ Lynch and Kilkelly in a small empirical study of New Zealand and Irish children's experiences of virtual hearings during the pandemic, found that the majority of children felt they did not fully understand what was happening during their live links to court.^28^

Harris and Goodfellow note that, ‘no government literature and limited data has been made available about the use of live links with defendants during COVID-19, and there is no specific information regarding children’.^29^ Indeed, an evaluation by HMCTS of the use of remote hearings during the pandemic, including their appropriateness with children was published in 2021. It makes no mention of children as a discrete group, similarly, no mention is made of youth courts (Clark, 2021).^30^

## Methods: Our Approach

The project commenced in November 2020, a period in which the GM region was under Tier 4 COVID-19 restrictions.^31^ Ethical approval was granted by Manchester Metropolitan University's Research Governance Committee.

We applied to HMCTS to gain approval to include legal professionals in the research. They would not grant permission for us to include Magistrates or Judges. We, therefore, undertook interviews with 14 legal professionals including seven Crown Prosecutors, three defence advocates and four Legal Advisors from the Youth Courts across the GM region. The interviews took place between June 2021 and November 2021. Interview guides included discussions about adaptations to courts, changes to individual roles, engagement with children, the use of video court hearings provision and short and long-term challenges for courts in a post-COVID-19 world. In addition, 77 interviews were carried out across the nine YOTs in GM between January and May 2021. We ran three participatory workshops involving 11 children aged between 16 and 17 who were working with a GM YOT. We undertook research in a Youth Offending Institute (YOI X) and a Secure Children's Home (SCH A) between March 2021 and January 2022. This involved 14 interviews with YOI X staff and a participatory workshop was facilitated on-site for six children aged between 16 and 17, each of whom had been in custody since the start of the pandemic. The research in SCH A involved telephone interviews with seven members of staff. In total, 21 children aged between 15 and 17 were either interviewed via Zoom or took part in three participatory workshops held on site.

### Analysis

Analysis was undertaken thematically, following the guidance from Braun and Clarke.^32^ All interviews were audio-recorded and transcribed. We devised a coding tree using NVivo software which led to the development of a list of codes reflecting the themes in the interview guides. All workshop discussions were audio-recorded and transcribed. Other written documentation such as flip chart exercises, were collated to assist with the analysis. We analysed these materials alongside the transcripts generated from the workshops.

## The Findings

Our analysis demonstrated that adaptations made to court processes throughout the pandemic were of direct consequence to child defendants, both in terms of how their cases were managed and how they personally engaged with the courts. In what follows, we seek to present a balanced consideration of the benefits and disadvantages of the adaptations, as they relate to the children.^33^

## Court Delays

GM legal professionals routinely commented that they felt that the Magistrates’ Courts had returned (almost) to normal relatively quickly, which they attributed to the variety of ways in which HMCTS had adapted its processes as part of its recovery plan. Key to this plan was the reduction of the back-log of cases to be heard. One solution implemented was to deal with blocks of cases represented by the same firm of defence solicitors. This meant that only a single defence advocate and prosecutor were needed for multiple trials, all of which were reviewed in advance to ensure they would be effective on the day of trial.

The trial work is a different kind of kettle of fish altogether because trial work is always more difficult because you’ve got a lot more factors in play. So, getting one particular defence firm and saying, ‘tell us all the cases that you have. We’ll re-review them. We’ll have these discussions. Is there any resolution?’ And then took those all into court. So, you just had one defence listed, one prosecutor, both had discussions about cases. They were all reviewed and ready to go. (Crown Prosecutor)

Whilst the summary work of Magistrates recovered relatively quickly, the case was not the same for youth work conducted in the Crown Court. These delays were routinely mentioned by legal professionals and professionals from YOTs. Our interviews with YOT colleagues detailed concerns about back-logs.

The issue is how long it's taking kids to get to court. And I don’t know whether that's a CPS issue or whether that's a GMP (Greater Manchester Police) issue. But, you know, kids are taking a ridiculous amount of time from the point of the offence to the investigation, the charge, the getting before a court. (GM YOT practitioner)

Others spoke of the increase in lengthy adjournments to cases involving children. Any form of delay was considered more problematic for children than for adults, due to the need for more immediate impact/intervention. The impact of delays to hearings was most keenly felt, according to GM professionals, to impact children's mental health. The prospect of having a case hanging over a child for an inordinate time period was recognised by professionals as detrimental to a child's ability to plan for the future and their engagement with services.

These kids that have got court pending over them for absolutely months and months. It's so life limiting. They can’t see anything apart from that date. Everything else around them, you’re trying to move them on, you’re trying to get them to do positive things and that's all they focus on is that date. (GM YOT practitioner)

The children we worked with in our project corroborate the concerns of professionals. For those children in the secure estate who described their literal journey through the courts to custody, the delays and adjournments to their court cases were a constant source of anxiety. The quote below from a 16-year-old boy serving a sentence in SCH A illustrates this.

So, last year January I got arrested and that. I got bailed and I got put on tag. I was meant to get sentenced in March last year. But then lockdown happened. Then they kept adjourning my whole case for like … my case went on for longer. See, I was on, like, tag for fourteen months and that. And they just kept adjourning it for fourteen months and then I got sentenced this year innit.

Professionals also expressed concern over the reliability of a child defendant's memory over time, and clearly delays to hearings exacerbated the situation:

I have had a couple of cases during the course of this, when you're asking a youth about something that happened eighteen months ago and invariably, they don't know the answers because it was eighteen months ago. … I definitely saw a few where I kind of thought, yeah, this is, definitely, going to hurt your case on the basis that it is just tougher to remember something that long ago. (Crown Prosecutor)

Increasing disaffection with the criminal justice system amongst child defendants was also highlighted as a potential consequence of delayed hearings and that whilst in the short term, there was a sense that children could not understand why they were only being punished now for something that had happened perhaps 2 years earlier, a longer-term impact was noted:

So the fact is that delaying all this activity is going to inevitably affect – or very likely affect – the reoffending rate, for example, because you are going to be working with a young person around an offence that might have been … well, it’ll feel like a different bloody universe to them, won’t it? (GM YOT professional)

However, for some children, a delay to their hearings meant that they had a longer period to demonstrate that they had not re-offended. This conferred upon them an advantage that would not have been taken into consideration during sentencing if their hearing had been earlier.

One defence solicitor was untroubled by there being a potential advantage to a child.

I think that young people have had to put up with an awful lot over the past 18, 19 months and if some of them have benefitted slightly because their case took many, many months to be finalised, well, so be it and that's the least that society can do for them. (Defence Solicitor)

## Prioritisation of Court Business

Except for custody hearings, there was a degree of uncertainty amongst GM professionals in terms of which youth cases should be further prioritised. Some professionals believed youth hearings were simply relisted in the same order as originally listed, whilst others felt that additional factors were considered throughout the listing process, including the seriousness of the offence which would determine the case being sent to the Crown Court. For instance, one Crown Prosecutor explained that since the Youth Courts hear most offences, s 20 assaults (i.e., Grievous Bodily Harm) and murders, they needed to be identified as a priority for sending to the Crown Court. However, another Crown Prosecutor felt that prioritising cases were of minor importance, given the potential impact on each individual child. It is well established that for child defendants, cases must be dealt with as soon as possible in order to maximise the impact on the child (CPS, 2020).^34^

To be honest it doesn’t really matter what the offence is because one youth defendant who is charged with a serious robbery and knife crime can just be as impacted as somebody who has never been before the court before and charged with simple criminal damage. It can be just as impactive on both of them for different reasons, so no, we don’t look at the offence and think right you need to go first. As long as you’re not in custody we’ll just try and get them through as quickly as we can. (Crown Prosecutor)

A lack of clarity over how to prioritise cases was most evident at the beginning of the pandemic, although it was acknowledged that as the pandemic progressed, so too did certainty over how cases should be prioritised.

I don’t think it was clear at the beginning and I think that’s why some things might have been missed. So, for example, whilst I was at home, I discovered a youth case that I thought fell into the urgent category and I contacted my immediate line manager and she disagreed with me that it did fall into that category. So, we then had to send it to the next level of management up who did agree that it did fall into that category. So, I think there was some confusion right at the beginning. (Legal Advisor)

The situation was arguably most frustrating for defence solicitors, who reported helplessness over being able to get their cases prioritised.

But we were ultimately in a situation where we couldn't force the court or the Crown to list a case. We sort of had to wait until we got a new day. All we were really doing was really pushing, certainly for some of the more vulnerable clients of ours, really pushing for the next court date and seeing what was happening. (Defence Solicitor)

Despite general uncertainty over prioritising, Crown Prosecutors took advantage of fewer court listings (and therefore more gained time) to focus on reviewing cases for two features in particular: (a) older cases and (b) low-level offences. For youth cases pending charge, prosecutors scrutinised their lists and prioritised work on the oldest cases. Youth cases were also reviewed for low-level offences, on the basis that expedient justice should be achieved where possible:

There was a lot of cases that you’d look at and think it was a low-level offence … I mean they weren't all low-level offences, but it came to light there's a lot of low-level offending here that's now getting delayed quite a lot. Particularly with the youth, the idea being simple expedient summary, to get things dealt with quickly so it's in their heads and has an impact as opposed to 12 months down the line. (Crown Prosecutor)

To this end, many of the legal professionals highlighted that “justice delayed is justice denied” in relation to not hearing youth cases quickly enough, summarised here by an interviewee from the CPS.

How can you punish your child for something that happened over a year ago? (Crown Prosecutor)

However, with that in mind, it was clear from the Crown Prosecutor interviewees that focussing on older and low-level offence cases was only possible because of the time gained from fewer court listings:

That wouldn’t have happened I don’t think because we wouldn’t have had the available staff, pre-pandemic. (Crown Prosecutor)

## Children's Engagement with the Courts

A discussion of children's engagement with the courts during the pandemic cannot proceed without a consideration of the impact of remote hearings conducted over the CVP, and indeed a good deal of research has started to emerge which focusses exclusively on this area.^35^

### Cloud Video Platform

The introduction of remote working in the courts was perhaps most clearly felt through the use of CVP. Legal professionals across GM highlighted both positive and negative aspects of integrating this technology into the courtroom. The difficulties, commented on by the majority of professionals, were most clearly felt in the area of interaction.

Children's capabilities to engage with the court over CVP were a key concern for interviewees.

… it’s difficult enough for an adult to kind of have a proper conversation virtually. We’ve all got used to it. But particularly for a young person and maybe for a young person that is struggling, the buy-in, the engagement, you know, there's just too many distractions. You take them into a room where they've only got the youth offending service to talk to, you may well get something out of them. Put them in their own house with a screen in front of them, and, you know, all these distractions, you're just not going to get the same kind of discussion with them. (Crown Prosecutor)

Children's capacity to concentrate on proceedings over CVP was raised by some interviewees who importantly noted that a bench of magistrates in the Youth Court would generally be able to respond appropriately to a child who is ‘drifting off’ whereas this may be missed over CVP. This potentially calls into question whether children are fully able to participate in their own hearings when conducted remotely.

So, we can stop and say, ‘Look, the system's not working, or the lawyer's not been able to contact with his client properly.’ We just say, ‘Right, we’ll stop it here.’ Does the youngster in the court understand what's going on now? And this is the point, you’re having to explain quite a lot. Whereas if you’re there in person, he can just tap you on the shoulder and say, ‘Look, do you want a break now while I explain everything to you?’ It's vitally important in the Youth Court. We have to ensure, you know, kids aren’t adults. They have a very, very short attention span. (Crown Prosecutor)

The ability to monitor children and their ability to participate in their own hearing feeds into the bigger issue of effective participation: the ability to understand and be involved in what is happening in court.^36^ Even before the outbreak of COVID-19, the use of live links in criminal court proceedings raised concerns that existing difficulties (e.g., mental health conditions, neuro-diverse conditions, cognitive impairments) were exacerbated.^37^ The literature indicates that criminal justice professionals in England and Wales felt that remote hearings prevented defendants from participating effectively, and the comments from the legal professionals across GM support this. When asked whether they had to make special efforts to ensure children were participating effectively in their remote hearings, no one felt that they had to adjust their advocacy or representation necessarily and did not appear to be aware of any guidance which dealt specifically with the use of remote hearings for children. This appears to be at odds with government assurances that ‘there is a range of guidance available regarding the use of remote hearings for children’.^38^

A clear benefit for advocates was that remote hearings meant they could get through more cases in one day than is possible with live hearings, requiring extensive travel between courthouses. Additionally, vulnerable professionals could continue working whilst maintaining their personal safety. This was especially significant for the CPS where there is a limited pool of prosecutors able to prosecute in the Youth Courts. CVP, therefore, enabled them to remain at capacity. However, despite these clear advantages, all GM professionals were very much in favour of live hearings. Indeed, interviewees were overwhelmingly of the opinion that if the child should be in court, so too should they.

We had this idea that if our clients were going to be brought to court, we ought to be present, and I had some sympathy for that, myself. So, yes, if the individual was in the court building, which almost certainly they were, then I think many of us felt we ought to be with them. (Defence Solicitor)

Particularly problematic, from the interviewees’ perspective, was those situations where both the defence and prosecution advocates might appear remotely, with the child being required to attend physically:

So, if the youth was arrested and remanded before the court for securement, they would still be in the courtroom and the defendant would still be present … But to have that level of change whereby there isn't a prosecutor in the room, potentially your defence advocate is not in the room with you either, yeah, it has to be, you imagine, something difficult for them to deal with. (Crown Prosecutor)

We have written in detail in earlier publications about the impact of COVID-19 on the ability of legal professionals to engage with children in the secure estate pre- and post-sentence.^39^ Indeed, staff from YOI X involved in the current project explained that at various points of the pandemic, defence solicitors were not allowed in the establishment and video links had to be set up to enable children to speak with their legal teams. They spoke of the canceling of trials and the delays in receiving post-court reports and how upsetting and frustrating this was for children, ‘because they didn’t know what was happening’. The technological advances brought about by the pandemic in the children's secure estate should not be underestimated. For instance, remote video conferencing for court hearings amongst the introduction of other modes of technology has dragged the secure estate into the twenty-first century. Staff at YOI X spoke with enthusiasm about the introduction of video link systems.

There was all new video link systems until they (the children) could speak to the solicitor because the solicitor was only allowed to go into court. They couldn’t come in here (YOI X). They put new video links in to speak to them (the children) before court cases. (YOI X Professional)

## Social Distancing Requirements

Some Youth Courts moved physically into another building or another court within the same building. This was largely to accommodate the greater number of participants in youth hearings compared to adult hearings, which was difficult in Youth Courts which were smaller rooms. It was interesting for inter-viewees to reflect on the impact for children having their case heard in the Youth Court whilst being situated in the physical space of an adult courtroom.

You know, in the old courts they were on a chair with their mum or their dad or their carer or social worker, and they’d be huffing and puffing. Just normal, like totally normal … Oh, this is ridiculous. F this and F that. And I think they’re probably quite scared when they walk in there [adult courtroom]. Some might say, not always a bad thing. But it's not what we’re meant … it's just not, you know, what we’re meant to do, you know. I think they probably are scared. So, they’re probably quieter. But, you know, on the flip side, they’re probably less engaging aren’t they because of it … like I say, you know, that intimidating courtroom, maybe their response has changed. Maybe they’re less engaging or engaged with the bench or the judge. (Crown Prosecutor)

Space limitations within the courts had an impact on children – and their families – in terms of having access to the courtroom. Social distancing requirements meant that the courts were not open to everyone and strict limits on the number of participants were enforced. This created issues for the families that wanted to support the child, and indeed the child themselves who wanted family support.

I think there was only maybe a couple of cases where people wanted someone in with them. I had one trial sort of further down the line when it was two defendants. It was in quite a small court, and we had probation, we had obviously two defence solicitors, a prosecutor, the legal advisor, the usher. And initially, they said, ‘Look, we can’t…’ They both had parents with them, and they were like, ‘We can’t have them both in the courtroom. So, there's nothing we can do’. And ultimately, the clients were really, really annoyed about that. And I think the parents were a bit annoyed as well. So, the court did actually facilitate it, so they could come in. *(*Defence Solicitor)

It was clear from discussions with YOT professionals that not only were children not receiving support from their families/carers due to space restrictions, the usual roles of YOT court staff were also severely curtailed due to the restrictions of numbers in the court rooms.

So, there were five barristers there for all the different defendants and they all went and sat in court. The usher said, ‘Oh no, we can’t have parents or YOT in because the barristers are in there’. I said, ‘Well can the barristers that aren’t dealing with that specific case not come out so that at least the parents can go in?’ and they wouldn’t. So, there was young people in court without YOT there and without parents there. (GM YOT professional)

Practice appears to have been variable between the Youth Courts, with some sticking rigidly to maximum occupancy levels, and others being more flexible.

Well, if there was more than one member of the youth offending team then we would only allow one of them in. I mean sometimes they bring both parents and a grandparent, so we had to say, ‘Look, we’re limited to numbers. Just one of you can come in. Do you want to decide between yourselves which one of you are going to come in with the young person?’” which is not ideal because if my daughter was in court, both me and my husband would want to be there. So, it was challenging and to be honest with you, we did break that rule sometimes. We made sure that people were socially distanced but we just maybe had to move people around the room just to make the distance because some families were very upset at not being able to come in. So, it worked. Sometimes we had to make it work. (Legal Advisor)

Social distancing for those children on remand in custody who were on trial during the pandemic, presented a range of challenges. For those who did not want to use CVP or could not (because their institution did not have the capabilities), were contending with myriad unfamiliar changes to how the court worked and what it looked like. For instance, the wearing of PPE and conversing with legal teams behind glass screens.

We had to wear masks, gloves, and aprons because if we didn't, we'd have to come back (to custody) and isolate. So, we had to wear the PPE, so we didn't have to isolate. All the court staff and that were always with the PPE equipment on. We was just always in the dock, but there was three rows, say one was on the end of the first row, and then one was in the middle of the second row, and one was on the other end of the third row. So, it was social distanced.’ (16-year-old boy SCH A).

Given that the children were in a secure setting and because of this not likely to be transmitters of COVID, they were put at risk of contracting and spreading it by their visits to court. The cumulative impact of this was evident in the discussions we had with children and staff in the secure estate who explained that children were infected through court visits and consequently, children in whole blocks of secure establishments had to isolate. Some children we spoke with had been required to isolate up to four times (10 days in solitary confinement) each time.

I think it was four days I was in court, that was the start of the trial. On the Thursday, I started getting symptoms. We had to stop the trial for two weeks and then restart it. I was tested here (secure establishment) and then they come back. Well, one of the (court) escorts phoned up on the Saturday and said that they've tested positive. So, we thought yeah, well I've got in then because I've been with them all week, and then it come back on the Sunday, and it was positive. (16-year-old boy SCH A).

## Discussion and Conclusions

The situation that arose throughout the pandemic – and the impacts created as a consequence raise a number of questions to consider about what the courts will (or indeed, should) look like in a post-COVID world. Amongst our participants, the short-term challenges related to the continuing impacts of delays and dealing with back-logs, although the overwhelming perspective from interviewees was that the back-log in the Youth Courts in GM was largely resolved. However, the national picture shows that the delays in the Youth Courts were a pre-existing problem that COVID-19 exacerbated. Indeed, the most recent youth justice statistics for England and Wales illustrate that the average time from offence to completion at court was 217 days, only four days lower than the previous year and well above pre-pandemic levels.^40^ This is concerning given that the evidence demonstrates the impact of delays on child defendants and especially on their mental wellbeing, quality of their evidence, and an increasing disaffection with the criminal justice system.^41^

Our research demonstrates that while courts moved swiftly to adapt their service delivery during the pandemic^42^ less is known about the quality and appropriateness of the service that children received. Gagnon and Alpern detail the creativity that courts drew on to respond expeditiously to the pandemic.^43^ However, in an English and Welsh context, we would argue that this creativity failed to account for a child's right to a fair trial. Utilising adult courts for child proceedings during the pandemic re-ignites the debate from the 2016 Taylor Review about the suitability of the Crown Court for cases involving children. We concur with the Taylor Review and its recommendation that cases involving children should only be heard in the Youth Court. While the protection of public health was the priority of the courts during the pandemic, we assert that HMCTS should have done more to ensure that children were not absorbed in the milieu of processes and legislation designed overwhelmingly for adult defendants.

The global response of courts to the pandemic serves as a reminder of the welfare origins of youth courts.^44^ In 2014, Lord Carlile's report stressed that Youth Courts ‘are only able to focus on the offence, and not the child and the wider circumstances contributing to their behaviour’.^45^ The combining of the Family Court with the Youth Court has long been mooted as a possible solution to better court outcomes for children however, Bateman suggests that a merger of the two could risk drawing more children into the court process and subsequent compulsory interventions.^46^ The Justice Select Committee of the UK Parliament in their report on Children and Young People in Custody, 2019–2020, recommended the introduction of direct recruitment to the Youth Magistracy, enabling Magistrates to specialise in the youth justice system from the outset (Magistrates currently have to sit 2 years in the adult court before being considered to sit in the Youth Court).^47^ Our findings demonstrate that legally qualified District Judges as opposed to Lay Magistrates were used increasingly in the Youth Courts during the pandemic. This decision was taken from a social distancing perspective rather than in the best interests of the child. The decision further emphasises the lack of a distinctive child-focussed response. Specialist advocacy for legal professionals working with children should be mandatory as part of legal qualifications and training. Children's effective participation in court is too often at risk, and a lack of specialism and understanding of children's needs is impacting children's experiences and outcomes.

In terms of CVP, the national literature indicates that even before the pandemic, concerns were raised about the impact of live links in exacerbating existing difficulties (e.g., mental health conditions, neurodiverse conditions, cognitive impairments), which may have prevented child defendants from participating effectively in their hearings and trials.^48^ These concerns are manifested within GM following the increased use of CVP. We therefore suggest that the current plans in the UK Parliament's Police, Crime, Sentencing and Courts Bill that will embed the expansion of virtual justice are premature. Professionals are overwhelmingly in favour of physical court appearances for children. A robust evaluation of remote justice is needed to assess its strengths and limitations with a particular focus on whether it is appropriate for use with children and in their best interests.

To conclude, the COVID-19 pandemic has laid bare the need for a full review of the functionality of Youth Courts across the globe. Our exemplar of the English and Welsh youth courts and our case study of GM's experience demonstrates an urgent need for renewed consideration of what support children need to effectively participate in court, and where and how children's cases should be heard. It has demonstrated that creativity is possible and creates a timely opportunity to review the evidence and think more radically about a welfare-based, trauma-informed court process for children. In their paper ‘Reimagining Youth Justice’, Gagnon and Alpern focus on the impact of COVID-19 and its potential for judicial policymaking and reform.^49^ They provide a quote from the Hon. Edwina Mendelson, Deputy Chief Administrative Judge for the Office of Justice Initiatives in New York State – ‘The word ‘reimagining’ rarely arises in the judicial context’.^50^ We contend that a re-imagination is exactly what is needed to address a child's right to a fair trial.

## Notes

1. GMYJUP is a formal, funded knowledge transfer partnership between Manchester Metropolitan University and each of the Greater Manchester Youth Justice Teams.

2. J.M. Baldwin, J.M. Eassey, and E.J. Brooke, (2020) ‘Court operations during the COVID-19 pandemic’, *Am J Crim Justice* (2020) 45(4): 743–758.

3. T. Sourdin, B. Li, and D.M. McNamara, ‘Court innovations and access to justice in times of crisis’, *Health Pol Technol* (2020) 9: 447–453.

4. A. Howe, ‘Courtroom access: Faced with a pandemic, the supreme court pivots’, SCOTUSblog 16 April 2020, available at: https://www.scotusblog.com/2020/04/courtroom-access-faced-with-a-pandemic-the-supreme-court-pivots/.

5. M. Harris and P. Goodfellow, *The Youth Justice System's Response to the COVID-19 Pandemic: Literature Review*, The Alliance for Youth Justice (2021), available at: https://www.ayj.org.uk/news-content/covid-project-literature-review.

6. Gov.uk, ‘Priority courts to make sure justice is served’, Press release 27 March 2020, available at: https://www.gov.uk/government/news/priority-courts-to-make-sure-justice-is-served.

7. Courts and Tribunals Judiciary, ‘Clarifying the application of well-established allocation principles in youth justice cases during the COVID-19 emergency’, 29 July 2020, available at: https://www.judiciary.uk/guidance-and-resources/clarifying-the-application-of-well-established-allocation-principles-in-youth-justice-cases-during-the-covid-19-emergency/.

8. HM Courts and Tribunals Service, (July 2020) ‘COVID-19: Overview of HMCTS response’, July 2020, available at: https://assets.publishing.service.gov.uk/government/uploads/system/uploads/attachment_data/file/896779/HMCTS368_recovery_-_COVID-19-_Overview_of_HMCTS_response_A4L_v3.pdf.

9. HM Courts and Tribunals Service, ‘COVID-19: Update on the HMCTS response for criminal courts in England & Wales’, September 2020, available at: https://assets.publishing.service.gov.uk/government/uploads/system/uploads/attachment_data/file/915493/HMCTS401_recovery_overview_for_crime_WEB.pdf.

10. Criminal Justice Joint Inspection, *The impact of the Covid-19 pandemic on the criminal justice system: A progress report*, January 2021, available at: https://www.justiceinspectorates.gov.uk/cjji/wp-content/uploads/sites/2/2021/01/2021-01-13-State-of-nation.pdf.

11. Ibid., at 22.

12. Ibid.

13. Ibid., at 26.

14. H. Smithson, P. Gray, D. Jump, S. Larner, and A. Nisbet, *The Youth Justice System's Response to the COVID-19 Pandemic*, Manchester Metropolitan University, (2021, June), COVID-19_and_Youth_Justice_Paper_1.pdf (mmu.ac.uk)

15. N. Lynch and T. Liefaard, ‘What is left in the “Too Hard Basket”? developments and challenges for the rights of children in conflict with the law’, *Int J Children's Rights* (2020) 28: 89–110.

16. See R. Adani and K. Dineen, ‘Discourses of childism: How COVID-19 has unveiled prejudice, discrimination and social injustice against children in the everyday day’, *Int J Children's Rights* (2021) 29: 353–370; N. Peleg, L. Lundy, and H. Stalford, ‘COVID-19 and children's rights: *Space* for reflection, *tracing* the problems and *facing* the future (Editorial)’, *Int J Children's Rights* (2021) 29: 255–259.

17. L.W. McHardy, ‘Preface’, *Juvenile Family Court J*(1998) 49(4): i.

18. P.L. Reichel & J.S. Albanese, ‘Comparing and delivering juvenile justice across the world’, in H. Kury, S. Redo, & E. Shea (Eds.), *Women and children as victims and offenders: Background, prevention, reintegration* (Switzerland: Springer International Publishing, 2016): 783–803.

19. United Nations Committee on the Rights of the Child (UNCRC), *General comment No. 24 (2019) on children's rights in the child justice system*, September 2019, available at: https://digitallibrary.un.org/record/3899429?ln=en.

20. Y. van den Brink, ‘Different but equal? Exploring potential catalysts of disparity in remand decision-making in the youth court’, *Soc Legal Stud* (2022) 31(3): 477–500.

21. See https://www.magistrates-association.org.uk/About-magistrates/Jurisdiction/Youth-court.

22. Crown Prosecution Service, ‘Coronavirus: Interim CPS Case Review Guidance – Application of the Public Interest COVID-19 crisis response’, 14 April 2020, available at: https://www.cps.gov.uk/legal-guidance/coronavirus-interim-cps-case-review-guidance-application-public-interest-covid-19.

23. Harris and Goodfellow, as above n. 5.

24. Criminal Justice Joint Inspection, *The impact of the Covid-19 pandemic on the criminal justice system: A progress report*, May 2022, available at: https://www.justiceinspectorates.gov.uk/cjji/wp-content/uploads/sites/2/2022/05/CJ-Covid-19-recovery-progress-report-web-2022.pdf.

25. HMCPSI, *CPS response to COVID-19: Dealing with backlogs The impact of COVID-19 on the CPS to 31 December 2020*, March 2021, available at: https://www.justiceinspectorates.gov.uk/hmcpsi/wp-content/uploads/sites/3/2021/04/2021-03-05-COVID-pressures-accessible.pdf.

26. See Harris and Goodfellow, as above n. 5.

27. See ibid.

28. N. Lynch & U. Kilkelly, ‘“Zooming In” on children's rights during a pandemic: Technology, child justice and COVID-19’, *Int J Children's Rights* (2021) 29(2): 286–304.

29. Harris and Goodfellow, as above n. 5 at 55.

30. J. Clark, *Evaluation of remote hearings during the COVID 19 pandemic Research report*, HM Courts and Tribunal Service, December 2021, available at: https://assets.publishing.service.gov.uk/government/uploads/system/uploads/attachment_data/file/1040183/Evaluation_of_remote_hearings_v23.pdf.

31. Tier 4 restrictions comprised a ‘stay at home’ directive allowing only essential activities.

32. V. Braun & V. Clarke, ‘Thematic analysis’, in H. Cooper, P. M. Camic, D. L. Long, A. T. Panter, D. Rindskopf, & K. J. Sher (Eds.), *APA handbook of research methods in psychology, Vol. 2: Research designs: Quantitative, qualitative, neuropsychological, and biological* (American Psychological Association 2012): 57–71.

33. S. Larner, A. Nisbet, and H. Smithson, The Youth Justice System's Response to the COVID-19 Pandemic. Research Paper 6: Court Adaptations. Manchester Metropolitan University, (2022, March), COVID-19_and_Youth_Justice_Paper_6.pdf (mmu.ac.uk)

34. Crown Prosecution Service, as above n. 22.

35. E.g. Lynch & Kilkelly, as above n. 28; Sourdin et al., as above n. 3; Baldwin et al., as above n. 2.

36. Joint Committee on Human Rights, *The Government's response to COVID-19: Human rights implication*, September 2020, available at: https://committees.parliament.uk/work/218/the-governments-response-to-covid19-human-rights-implications/publications/.

37. Ofsted, *SEND: Old issues, new issues, next steps*, 16 June 2021, available at: https://www.gov.uk/government/publications/send-old-issues-new-issues-next-steps#:∼:text=A%20report%20about%20the%20experiences,over%20the%20last%2010%20years.

38. House of Commons Select Committee, *Children and Young People in Custody (Part 1): Entry into the youth justice system: Government Response to Committee's Twelfth Report of Session 2019–21*, 3 February 2021, available at: https://committees.parliament.uk/publications/4636/documents/46905/default/.

39. H. Smithson, D. Jump, and A. Nisbet, The Youth Justice System's Response to the COVID-19 Pandemic. Research Paper 6: Court Adaptations: The Impact on Youth Custody. Research paper 7, COVID-19_and_Youth_Justice_Paper_7.pdf (mmu.ac.uk)

40. Youth Justice Board, ‘Youth Justice Statistics, 2021–2022’, 23 January 2023, available at: https://www.gov.uk/government/collections/youth-justice-statistics.

41. G. Hunter, C. Ely, C. Robin-D’Cruz, and S. Whitehead, *Time to get it right: Enhancing problem-solving practice in the Youth Court* (Centre for Justice Innovation, 2020).

42. Gov.uk, as above n. 6.

43. A. Gagnon & S. Alpern, ‘Reimagining youth justice: How the dual crises of COVID-19 and racial injustice inform judicial policymaking and reform’, *Juvenile Family Court J* (2021) 72(2).

44. McHardy, as above n. 17; Reichel & Albanese, as above n. 18.

45. A. Carlile, *Independent Parliamentarians’ Inquiry into the operation and effectiveness of the Youth Court* (Michael Sieff Foundation: London, 2014) ix, available at: http://michaelsiefffoundation.org.uk/content/inquiry_into_the_operation_and_effectiveness_of_the_youth_court-uk-carlile-inquiry.pdf.

46. T. Bateman, *Bridging the care-crime gap: Reforming the youth court?* (NAYJ, 2021).

47. House of Commons Select Committee, as above n. 38.

48. Van den Brink, as above n. 20.

49. Gagnon & Alpern, as above n. 43.

50. Mendelson, as cited in ibid. 6.

